# A fatal case report of antibody-dependent enhancement of dengue virus type 1 following remote Zika virus infection

**DOI:** 10.1186/s12879-021-06482-0

**Published:** 2021-08-04

**Authors:** Ashley N. Bonheur, Sarah Thomas, Sara H. Soshnick, Emily McGibbon, Alan P. Dupuis, Rene Hull, Sally Slavinski, Paula E. Del Rosso, Don Weiss, Danielle T. Hunt, Megan E. McCabe, Amy B. Dean, Rebecca Folkerth, Anne M. Laib, Susan J. Wong

**Affiliations:** 1 Division of Pediatric Critical Care Medicine, The Children’s Hospital at Montefiore/Albert Einstein College of Medicine, Bronx, NY USA; 2 Office of the New York City Chief Medical Examiner, New York, NY USA; 3 New York City Department of Health and Mental Hygiene, Queens, NY USA; 4 Wadsworth Center, New York State Department of Health, Albany, NY USA

**Keywords:** Dengue virus, Zika virus, Antibody dependent enhancement, Avidity assay, Case report

## Abstract

**Background:**

Dengue virus (DENV) is endemic in many parts of the world. Antibody dependent enhancement (ADE) in DENV infections occurs when a person with primary immunity is infected by a second, different DENV strain. Antibodies to Zika virus (ZIKV), which emerged in the Western Hemisphere in 2015, are cross reactive with DENV and theoretically could provoke ADE in a DENV naïve individual.

**Case presentation:**

DENV infection was suspected in a child who had recently returned from a one-month stay in the Dominican Republic. The child presented with fever, vomiting, abdominal pain, and in hypovolemic shock. Volume and pressor resuscitation were unsuccessful, and the child died less than 24 h after hospitalization. Laboratory results suggested an early acute first DENV infection since serum, plasma, and spinal fluid had DENV1 detected by polymerase chain reaction (PCR), yet the serum lacked IgG antibodies to DENV nonstructural protein 1 (NS1) of all four DENV serotypes. This acute DENV infection occurred in the presence of a remote ZIKV infection as determined by antibodies to ZIKV NS1 envelope by multiplex microsphere immunoassay and an exceptionally high plaque reduction neutralization titer to ZIKV. ZIKV IgG avidity index was high, confirming a past infection. DENV1 RNA was detected in all ten organs and tissues examined by PCR. The severe and fatal complications reported here suggest that a remote ZIKV infection may provoke an exaggerated immune response leading to hypovolemic shock when primarily infected by DENV1.

**Conclusion:**

We report the first known patient in the United States with a rapidly progressive and fatal case of travel-associated DENV in which prior exposure to ZIKV likely played a role in triggering an ADE phenomenon. This association of prior ZIKV immunity and subsequent new dengue infection is a worrisome phenomenon and an important contribution to the body of knowledge on immunity to flaviviruses.

## Background

Dengue infection (DENV) is the most common mosquito borne viral disease in the world [1]. DENV is a positive sense RNA virus in the *Flaviviridae* family, genus flavivirus, that occurs as one of four serotypes [2]. Dengue viruses are closely related to Zika virus (ZIKV): both are members of the *Flaviviridae* family and have immunologic cross-reactivity due to their amino acid homology [3, 4]. In children, DENV often causes an asymptomatic or mild and nonspecific illness 2–7 days following the bite of an infected *Aedes* mosquito. A very small proportion of infections will have the severe complications of dengue hemorrhagic fever (DHF) or dengue shock syndrome (DSS).

Severe complications of DENV infection may occur with a first infection (primary infection) but are more frequent when a patient is infected with a second DENV of a different serotype often due to a phenomenon known as antibody dependent enhancement (ADE) [5, 6]. In ADE, cross-reactive antibodies to pre-membrane and envelope proteins from the primary DENV virus serotype allow binding of the second DENV virus-IgG immune complexes by Fc-receptors on monocytes, facilitating virus transport across cell membranes and increased viral replication. DENV non-structural protein 1 (NS1) causes direct damage to endothelial cells resulting in plasma leakage [6, 7]. In children, a second DENV infection has a ten-fold higher risk of DHF and DSS than a primary infection. Infants less than one year old, who have acquired DENV antibodies via transplacental passage from mothers with a history of previous DENV infection have a higher risk of DHF and DSS than infants born to mothers who have never had DENV [8].

While limited local transmission of DENV has been detected in Hawaii, Florida, and Texas, the majority of DENV infections among United States residents are acquired during travel to visit friends or relatives in endemic areas including Southeast Asia, Latin America, and the Caribbean [9–12]. Florida, New York, and California report the highest number of cases each year in the continental United States and numbers are reflective of global activity with peak years resulting in more imported cases [12]. The largest number of cases in New York City occurred in 2010 (N = 144), the year of a large DENV outbreak in Latin America. That year, three deaths were reported, which were the last reported DENV deaths in New York City until the case we describe here.

The ZIKV pandemic affected much of South, Central, and Latin America from 2015 to 2017, but may have been introduced into Brazil as early as 2013 [13]. This has prompted concern that DENV infection following a ZIKV infection may result in a similar ADE phenomenon as with a heterotypic DENV serotypes. Prospective studies to determine severity of dengue after Zika are planned in central America (SW personal communication with Steve Waterman, Centers for Disease Control and Prevention, September 6, 2019). We present the case of a child who had DHF/DSS and laboratory evidence of probable ADE as a result of a prior ZIKV infection. Currently, there are no published reports of pediatric mortality as a consequence of ADE following a ZIKV infection.

## Case presentation

### Patient’s clinical course

**Fig. 1:**
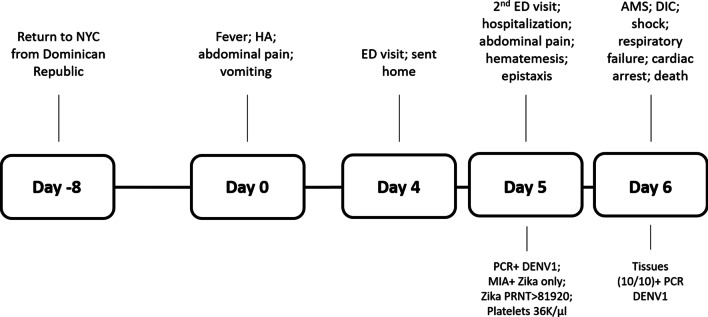
Patient timeline. *HA* headache, *AMS* altered mental status, *DIC* disseminated intravascular coagulation

A previously healthy, United States born, Hispanic, school-aged female known to have sickle-cell trait presented to a New York City emergency department (ED) in August 2019 with a 4-day history of fever (maximum-38.4 °C), headache, abdominal pain, and vomiting. The patient was diagnosed with acute gastroenteritis and discharged home. She presented to a second emergency department 14 h later prompted by worsening abdominal pain and episodes of epistaxis, hematemesis, and black tarry stools. The patient had recently returned from a one month visit to the Dominican Republic two weeks prior to presentation (Fig. 1), during which it was reported that her cousin was hospitalized with dengue fever. Upon arrival to the second ED, she was afebrile (initial-36.6 °C, maximum-37.3 °C) with a pulse of 122 beats/min, blood pressure of 122/62 mm Hg, and a respiratory rate of 21 breaths/min. Her oxygen saturation was 85% in room air, which increased to 98% with 100% oxygen delivery via non-rebreather. Significant findings on physical examination were skin pallor, cold extremities with a prolonged capillary refill, weak pulses, waxing and waning consciousness, and a distended abdomen with rebound tenderness. The patient was determined to be in hypovolemic and septic shock. Broad-spectrum antibiotics were administered, and resuscitation efforts were initiated.

Timeline of patient risk from travel to onset of symptoms, ED visit, hospitalization and death. Key clinical factors are listed above, and laboratory findings are presented below the days post onset.

Initial laboratory results were significant for hyponatremia (130 mEq/L), hyperkalemia (5·5 mEq/L), and a severe metabolic acidosis (bicarbonate 6 mEq/L, venous pH < 6·8 and lactate level 14·2 mmoL/L). The white blood cell count was elevated at 32·9 k/µL with 61% neutrophils, 34% lymphocytes, and 3% immature granulocytes. The patient was anemic and thrombocytopenic with a hematocrit of 26·6% and a platelet count of 36 k/µL. Poikilocytosis (3 +), burr cells (3 +), anisocytosis (1 +), macrocytosis (1 +), spherocytosis (1 +), and schistocytosis (1 +) were noted, but no sickling. A malaria antigen test was negative and no parasites were noted on thick or thin blood smears. Blood urea nitrogen was 21 mg/dL and creatinine was 0·8 mg/dL, both elevated for the patient’s age. Liver enzymes were significantly elevated with an aspartate aminotransferase of 2137 U/L and alanine transaminase of 744 U/L, and her serum albumin was subnormal at < 2·0 g/dL. Coagulation studies were concerning for disseminated intravascular coagulation (DIC) with prolongation of her prothrombin time (PT) to 32·2 s, partial thromboplastin time (PTT) to 95·1 s, and an Internal Normalized Ratio (INR) of 3·2. Lactic acid dehydrogenase (4,171 U/L) and uric acid (9·6 mg/DL) were also abnormal. An initial portable chest radiograph revealed clear lungs and a computerized tomography (CT) scan with contrast of the abdomen was obtained due to the concern for an intraabdominal process. The CT scan revealed significant ascites, bilateral pleural effusions, a small pericardial effusion, thickening of the bowel, a collapsed inferior vena cava, lack of enhancement of the liver and spleen, and delayed nephrograms, all consistent with hypovolemic shock.

In the pediatric intensive care unit, the patient was intubated for altered mental status and worsening respiratory failure. Central venous and arterial access was obtained, and despite fluid resuscitation with both crystalloids and colloids, the patient became increasingly hypotensive with a narrow pulse pressure, requiring multiple vasopressors for blood pressure support. Repeat labs revealed worsening hyperkalemia (9·0 mEq/L) with refractory acidosis (bicarbonate < 5 mEq/L, arterial lactate of 17 mmol/L). A repeat chest radiograph revealed increasing pleural effusions consistent with worsening capillary leak. Fulminant DIC developed exhibited by bleeding from the mouth, nares, mucosa, and the central line site. Repeat laboratory tests were consistent with a rapidly progressive coagulopathy with a PTT of > 300 s, PT of 53 s, INR of 6·0, fibrinogen level of 55 mg/dL, and D-Dimer of 2·43 μg/mL. The patient remained in catecholamine and fluid refractory shock despite the administration of packed red blood cells and fresh frozen plasma. Twelve hours after presentation to the second ED the patient suffered an asystole cardiac arrest. A bedside echocardiogram showed no evidence of pericardial tamponade. Despite all efforts the patient expired. Postmortem genetic testing confirmed the patient was heterozygous for hemoglobin AS (sickle cell trait).

### Findings at autopsy

External examination revealed no rash. The oral and conjunctival membranes were pale. There were pleural and pericardial effusions, as well as a small amount of peritoneal fluid. The lungs were congested and edematous, with numerous, scattered, mucosal petechial hemorrhages along all bronchial branches. The epicardial surface also had scattered petechial hemorrhages and there were patchy, subendocardial hemorrhages of the left ventricle. The stomach mucosa was markedly erythematous, with associated dispersed patches of finely raised, lymphoid nodular stippling. The stomach, small and large intestines contained a small amount of blood. Sparse tissue hemorrhages were present within the peritoneal cavity and surrounded the adrenal glands, small intestine, and posterior uterus. There were no intraparenchymal adrenal gland hemorrhages. The mesenteric tissues had marked lymphadenopathy with associated patchy hemorrhagic congested areas. The spleen was enlarged and indurated, with slightly pale-pink, capsular discolorations. The liver parenchyma was congested. A discrete focus of acute parenchymal hemorrhage was noted within the pancreatic head, subjacent to the sphincter of Oddi. Throughout the body cavity, within the visceral fascial planes, there was marked subcutaneous emphysema. The brain was edematous and the leptomeninges were congested primarily adjacent to the superior sagittal sinus, in the distribution of the arachnoid granulations. The cerebrospinal fluid was bright red tinged.

**Fig. 2:**
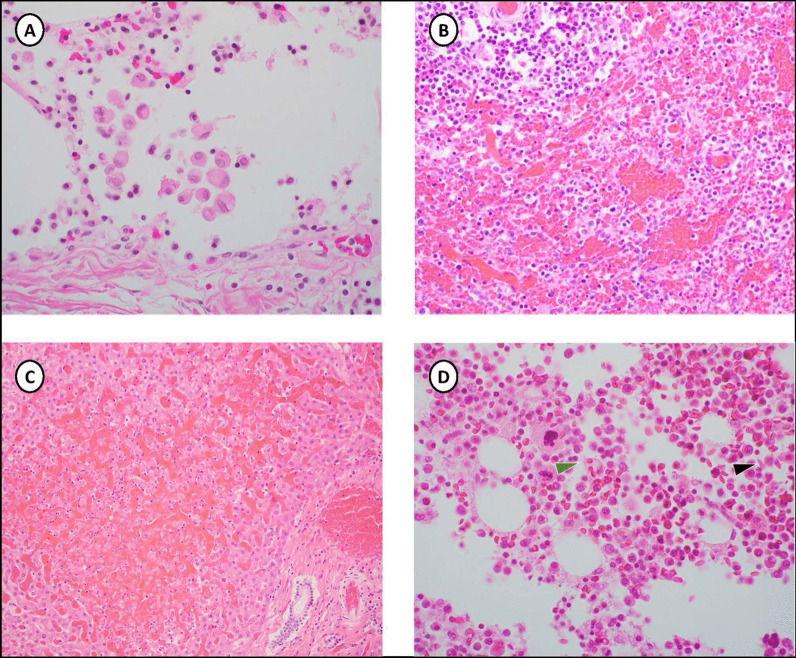
Histopathology- Hematoxylin and eosin staining (magnification 400×) **A** Lung with alveolar macrophages containing intact erythrocytes (hemophagocytosis). **B** Spleen with markedly congested sinusoids. **C** Early, focal liver necrosis and sinusoidal hemorrhage. **D** Section of bone marrow with histiocyte containing intact erythrocytes (hemophagocytosis, green arrow) and red blood cell sickling (black arrow)

Histopathologic examination was concordant with the gross findings of tissue and mucosal hemorrhage. Hemophagocytosis was seen in the lungs and bone marrow, there was marked sinusoidal congestion of the spleen, and red blood cell sickling (Fig. 2). Hepatocyte necrosis was present, and the kidneys showed many multifocal areas of tubule mineralization and marked edema of the glomeruli. The stomach had dense mucosal lymphocytic infiltration and prominent reactive lymphoid follicles. Hemophagocytosis was marked within intra-alveolar macrophages and among scattered bone marrow histiocytes. Sections of heart tissue did not show evidence of myocarditis.

### Laboratory diagnosis

Whole blood, serum, cerebrospinal fluid **(**CSF**)**, and fresh frozen organ tissue samples were collected 5- and 6-days post onset of symptoms and at autopsy (day 7). Molecular diagnostic testing was performed using an FDA approved, CDC developed 1-step real-time RT-PCR assay that detects and differentiates between the DENV serotypes 1, 2, 3, and 4 [14]. Real-time RT-PCR assays for the detection of ZIKV and Chikungunya virus were also performed [15, 16]. DENV1 was detected in whole blood, serum, cerebrospinal fluid, and in frozen tissue from ten organs (heart, lung, liver, spleen, kidney, adrenal gland, intestine, brain, stomach, and lymph node). The lowest cycle threshold (corresponding to the highest level of RNA) value was found in the liver, the tissue with the most marked pathologic changes (Table 1). The highest cycle threshold value was found in the heart where no significant histopathologic changes were detected. RT-PCR for ZIKV and Chikungunya were negative.

**Table 1:** Dengue PCR results and Cycle thresholds

Sample type	Days post onset	Dengue 1 Real-time PCR Cycle threshold
Whole blood*	5	34.83
Whole blood*	7	26.43
Serum**	6	28.17
CSF	7	28.88
Tissue A-Mesentery #1	7	34.83
Tissue B-Kidney	7	28.77
Tissue C-Thymus	7	24.19
Tissue D-Pancreas	7	28.92
Tissue E-Mesentery #2	7	28.60
Tissue F-Liver	7	19.04
Tissue G-Lung	7	21.80
Tissue H-Heart	7	30.64
Tissue I-Lymph Node	7	30.31
Tissue J-Spleen	7	24.77

*Screened for Zika and chikungunya viruses

**Screened for chikungunya virus

Commercial laboratory antemortem serology testing found Dengue Fever IgM = 1·73 (reference ranges: negative, < 0·80, equivocal 0·8–1·09, positive ≥ 1·10) and IgG = 7·59 (reference ranges: negative, ≤ 1·65, equivocal 1·66–2·83, positive ≥ 2·84). Commercial assays for dengue were developed and cleared by FDA prior to arrival of Zika in the Western Hemisphere. The available DENV IgG assays are based on a broadly cross-reactive envelope protein and cannot differentiate between *Flaviviruses.* The above IgM result is consistent with acute DENV infection whereas the IgG result, as shown by further testing below, is likely explained by cross reaction from previous Zika infection.

**Fig. 3:**
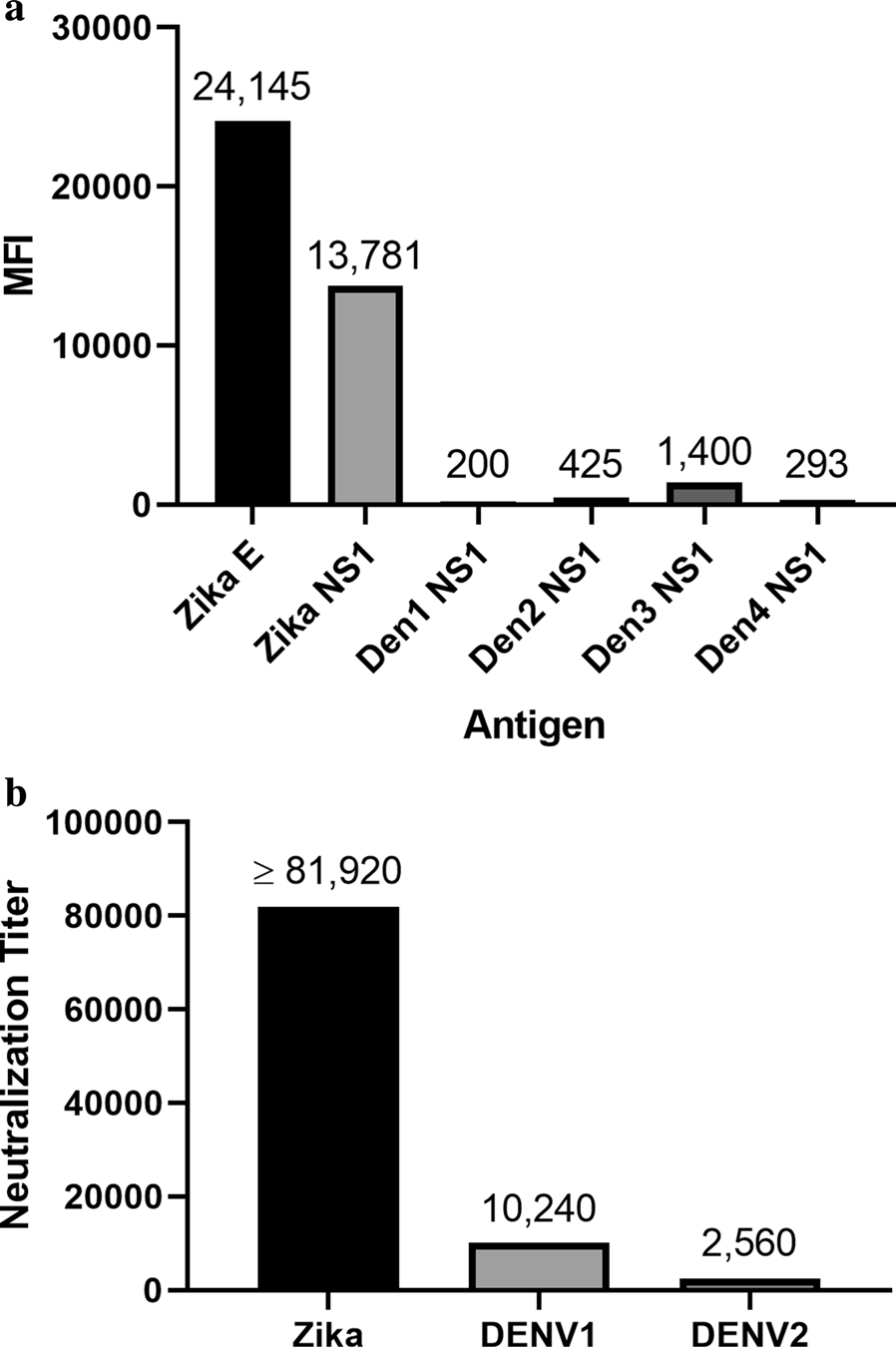
**a** Multiplex microsphere immunoassay measurement of total antibodies to Zika envelope, Zika NS1, and the NS1 proteins of all four dengue serotypes are presented as the median fluorescence intensity (MFI) of 100 beads counted for each antigen coated beads on the Y axis. The target antigens are reported on the X axis. This multiplex analysis demonstrates past Zika infection. The dramatically lower level of antibodies to dengue NS1 proteins makes a past dengue infection unlikely. **b** Shows the virus being neutralized. Exceptionally high PRNT to Zika indicates past infection. The lower PRNT to DENV1 and DENV2 may indicate cross reaction of the Zika antibodies recognizing dengue since it is the envelope protein being neutralized

At the Wadsworth Center total antibodies (IgG + IgA + IgM) to ZIKV and DENV were evaluated using a suspension phase microsphere immunoassay [17]. The test was developed, validated, and approved for clinical use by the New York State’s Clinical Laboratory Evaluation Program (CLEP). Serum collected 5 days and 6 days after onset had a high level of antibodies to Zika envelope and Zika NS1 yet lacked significant detectable antibodies to NS1 of the four dengue serotypes, thus ruling out a previous dengue infection (Fig. 3a).

Cross species plaque reduction neutralization tests (PRNT 90) were performed in Vero cells and showed an exceptionally high titer to ZIKV (> 81,920) and lower titers to DENV1 (10,240) and DENV2 (2560) (Fig. 3b) [18]. Viral culture in Vero cells was attempted using plasma, spinal fluid and autopsy tissue. After two weeks no viral growth was detected.

An avidity index (AI) is a measure of IgG binding affinity that was developed to determine the amount of time passed since a flavivirus infection [19–21]. IgG antibodies mature over time becoming more selective through a process akin to natural selection [22]. Mutations in antibody-producing B lymphocytes create a pool from which non-neutralizing antibody producers are deselected [8, 23]. IgG avidity increases in a nearly linear manner achieving AI of about 50% at 6 months post infection. After 6 months IgG avidity will continue to increase sometimes reaching 80–90%; however, exposure to another flavivirus will also boost the AI to these levels. The Wadsworth Center has developed, validated, and received CLEP approval for an IgG avidity assay. Most adults living in dengue endemic areas have IgG avidity indices to dengue NS1 proteins in the 75–95% range [21]. The IgG AI for this patient was 85% to Zika envelope and 55% to Zika NS1, indicative of remote Zika infection > than 6 months prior to specimen collection. An IgG avidity to dengue was not performed for this patient since there were no significant levels of detectable IgG antibodies to the dengue NS1 proteins in the early acute infection.

## Discussion and conclusions

We describe a case of fatal DHF/DSS due to DENV1 in a school-aged child whose exposure occurred in the Dominican Republic. Serologic evidence confirmed a prior ZIKV infection, but not a prior DENV infection which, along with the severity of the illness, was consistent with ADE.

Research has shown that the risk of DHF/DSS is elevated in the presence of preexisting DENV antibodies of a different serotype [8, 24, 25]. Since ZIKV antibodies are known to cross-react with DENV, it has been hypothesized that individuals with ZIKV immunity may be at risk for ADE when then exposed to DENV. Antibodies to ZIKV likely have a decreased capacity to neutralize DENV, but still have binding affinity. Immune complexes composed of ZIKV antibodies attached to DENV would have the ability to fix complement and bind to cell surface Fc receptors enabling virus entry into phagocytic cells. Enhanced viral replication ensues leading to more severe manifestations of disease [8].

Data from DENV outbreaks in Cuba in 1977 and 1981 support the ADE hypothesis. The 1977 outbreak was caused by DENV1 and resulted in an estimated 500,000 cases [26]. In 1981, DENV2 was the responsible strain and there were over 10,000 severe illnesses with 101 deaths in children [26]. A subsequent serosurvey found that the prevalence of DENV1 antibodies following the 1977 outbreak was 44.5%. Among 124 severely ill children who survived the 1981 outbreak, 98% had antibodies to both DENV1 and 2, supporting the notion that the presence of those antibodies placed children at a higher risk for severe disease [24]. Furthermore, no child aged 1–2 years old (born after the 1977 outbreak) was hospitalized during the 1981 outbreak [26].

Additional support for the ADE phenomenon comes from a safety and efficacy of trial of Dengvaxia, a live attenuated tetravalent dengue vaccine, that was studied in the Philippines [27]. Analysis of the data revealed that children less than 9 years of age without previous DENV immunity who received vaccine were found to have an increased risk of hospitalization for severe disease [28]. Data from a Nicaraguan pediatric cohort has further suggested that dengue ADE occurs within a narrow range of pre-existing antibody titer which places children at the highest risk for severe illness in the immediate years following their initial DENV infection or vaccination [25].

Hemophagocytic lymphohistiocytosis (HLH), an immune dysregulation disorder featuring macrophage destruction of erythrocytes and uncontrolled cytokine production [29], is known to occur in DENV infections [30]. The patient described here met 5 of 8 HLH 2004 classification criteria: fever, splenomegaly, bicytopenia, hypofibrinogenemia, and bone marrow hemophagocytosis [31]. The features of HLH are difficult to distinguish from DHF/DSS and there is considerable diagnostic overlap with sepsis [32]. The interrelationship between ADE and HLH is unknown. Whether HLH complicated the patient’s course cannot be determined.

After ZIKV arrived in the Western Hemisphere in 2015–2016, concerns arose that DHF/DSS could appear as a complication of antecedent ZIKV infection, particularly in children. Animal studies have shown that newborn mice born to mothers with ZIKV immunity had increased mortality when infected with DENV [33] and macaques with ZIKV immunity have increased viral loads and pro-inflammatory responses (without increased disease severity) when challenged with DENV2 [34]. Epidemiology studies, however, were unclear on whether ZIKV immunity increased DENV severity [35, 36], however, an analysis of the aforementioned Nicaraguan children cohort following the 2019–2020 DENV2 outbreak has confirmed that prior ZIKV increases the risk for severe DENV2 [37]. The authors hypothesized that prior ZIKV could provoke ADE in other DENV serotypes, as occurred with this child [37].

The patient’s family reported travel to the Dominican Republic during the summers of 2016 and 2017, a time of peak ZIKV transmission in that country. It is likely the ZIKV exposure in this child occurred during one of these visits leading to the development of ZIKV antibodies that were responsible for enhancing the immune response to DENV1 acquired in 2019. We cannot entirely rule out the additional immunologic effects of an antecedent DENV infection acquired during a prior trip to the Dominican Republic, however, the antibody titers strongly implicate ZIKV ADE. While sickle cell disease (hemoglobin SS and SC) has been found to increase mortality to DENV the same has not been reported for persons with sickle cell trait [38, 39]. It is conceivable that in the face of hypoxemia and acidosis, the patient’s sickle hemoglobin contributed to the disease progression.

DENV, and other non-endemic arboviruses, may not be considered by providers in the United States and familiarity with WHO guidelines is warranted in communities with frequent travel to flavivirus endemic countries [40]. Providers evaluating DENV compatible illnesses should obtain a detailed history including birthplace and recent travel. Early detection of signs of shock or other complications attributable to ADE DENV can help prevent a fatal outcome. DHF/DSS patients require acute intensive medical care and aggressive fluid resuscitation is recommended based on a rising hematocrit, which did not occur in this child [40]. Treating DHF/DSS differs from the management of septic shock from other etiologies as early fluid resuscitation is part of the mainstay of the latter’s treatment. Without a high index of suspicion and or rapid assays to diagnose infection as well as prior immunity to DENV and or ZIKV, prompt and appropriate care remains a challenge.

We report the first known occurrence in a United States resident of DENV ADE precipitated by prior ZIKV immunity. As new and existing arboviruses emerge/reemerge it is important to understand how they may interact and the implications this has for both vaccine development and clinical care. Further research is required to decipher the immune responses to DENV, ZIKV, and other flaviviruses and to develop accurate rapid assays for clinical diagnosis.

## Data Availability

Data sharing is not applicable to this article as no datasets were generated or analysed during the current study.

## Abbreviations

DENV: Dengue virus
ADE: Antibody dependent enhancement
ZIKV: Zika virus
PCR: Polymerase chain reaction
NS1: Nonstructural protein 1
DHF: Dengue hemorrhagic fever
DSS: Dengue shock syndrome
HA: Headache
AMS: Altered mental status
DIC: Disseminated intravascular coagulation
CT: Computerized tomography
CSF: Cerebrospinal fluid
MFI: Median fluorescence intensity
CLEP: Clinical laboratory evaluation program
RT: Real-time
PRNT: Cross species plaque reduction neutralization tests
HLH: Hemophagocytic lymphohistiocytosis
AI: Avidity index
